# Atrial natriuretic peptide increases the albumin permeability of mouse skeletal muscle microcirculation by the caveolae-mediated transcellular pathway

**DOI:** 10.1186/1471-2210-11-S1-P16

**Published:** 2011-08-01

**Authors:** Wen Chen, Birgit Gaßner, Sebastian Börner, Michaela Kuhn

**Affiliations:** 1 Institute of Physiology, University of Würzburg, Germany

## Background

Cardiac atrial natriuretic peptide (ANP) is an essential physiological regulator of arterial blood pressure and intravascular volume. Unlike other "natriuretics", which reduce interstitial fluid volume with little change in plasma volume, ANP has important extrarenal, endothelial actions that enable it to reduce plasma volume preferentially. Thus ANP, via its guanylyl cyclase-A (GC-A) receptor and cyclic GMP formation, enhances microvascular endothelial permeability to albumin, ultimately moving plasma fluid into interstitial pools. However, the cellular pathways mediating this effect are unknown.v Here we investigated the role of caveolae-mediated endothelial albumin transcytosis in the mechanism of increased permeability induced by ANP.

## Methods and results

Intravital microscopy showed that the vasodilating effect of ANP on cremaster precapillary arterioles was similar in mice with endothelial-restricted deletion of GC-A (EC GC-A KO) and in control mice. In controls, ANP stimulated the extravasation of fluorescent albumin from cremaster postcapillary venules. This permeability effect was abolished in EC GC-A KO mice and in mice with ablated caveolin-1 (cav-1), the caveolae scaffold protein. Concomitantly, the ANP-induced acute systemic loss of intravascular fluid volume was abolished in EC GC-A KO and Cav-1^-/-^ mice. In vitro, ANP increased fluorescent albumin uptake and transcytosis in cultured microvascular endothelial cells without affecting transendothelial electrical resistance. ANP stimulation of albumin uptake was abolished in GC-A- or cav-1-deficient endothelia. Lastly, enrichment of caveolae from mouse lung together with western blot analyses demonstrated that a population of GC-A receptors colocalizes with Cav-1 in caveolae microdomains.

## Conclusion

ANP exerts two types of microvascular effects. It acts as endothelium-independent vasodilatator. In addition the peptide mildly stimulates transendothelial caveolae-mediated vesicular albumin transport through GC-A localized in membrane rafts and caveolae. This effect is independent from the vasodilating action. The ANP-mediated communication between the heart and the microcirculatory endothelium is essential for the maintenance of intravascular volume homeostasis.

